# Chinese Herbal Medicine Ameliorated the Development of Chronic Kidney Disease in Patients with Chronic Hepatitis C: A Retrospective Population-Based Cohort Study

**DOI:** 10.1155/2019/5319456

**Published:** 2019-11-21

**Authors:** Che-Pin Chang, Yuan-Chih Su, Mei-Chen Lin, Sheng-Teng Huang

**Affiliations:** ^1^Department of Chinese Medicine, China Medical University Hospital, Taichung, Taiwan; ^2^Management Office for Health Data, China Medical University Hospital, Taichung, Taiwan; ^3^School of Chinese Medicine, China Medical University, Taichung, Taiwan; ^4^An-Nan Hospital, China Medical University, Tainan, Taiwan; ^5^Chinese Medicine Research Center, China Medical University, Taichung, Taiwan; ^6^Research Center for Chinese Herbal Medicine, China Medical University, Taichung, Taiwan; ^7^Cancer Research Center for Traditional Chinese Medicine, Department of Medical Research, China Medical University Hospital, Taichung, Taiwan

## Abstract

Chronic kidney disease (CKD) is a serious complication affecting patients with chronic hepatitis. The effectiveness of CHM for the prevention of CKD in hepatitis patients remains unclear. Therefore, we conducted a retrospective cohort study to investigate the effectiveness of CHM in preventing the development of CKD in hepatitis patients. From a subdataset of the Taiwan National Health Insurance Research Database (NHIRD), we included 19,409 patients newly diagnosed with hepatitis B and hepatitis C between the years 2000 and 2010. After exclusion criteria and 1 : 1 propensity score matching process, we compared demographic factors, comorbidities, and correlated drugs between the CHM and non-CHM cohorts. Statistical analysis was applied to evaluate the differences in characteristic distributions and to compare the cumulative incidence of CKD between the CHM and non-CHM cohorts. This study showed that the patients suffering from hepatitis C with CHM treatment more than 90 days as an adjuvant therapy combined with western medical treatment modalities exhibited a decreased risk of developing CKD (hazard ratio (HR) = 0.40, 95% confidence interval (CI) = 0.21–0.76, *p* value <0.01). The Kaplan–Meier curve revealed a lower cumulative incidence rate of CKD (*p* value = 0.004) for the CHM cohort. For further reference, we herein offer the ten most frequently prescribed single herbs and herbal formulas; as such, *Salviae miltiorrhizae* and Jia-Wei-Xiao-Yao-San were the most commonly prescribed single herb and formula, respectively. This nationwide retrospective cohort study provides evidence that CHM is an effective adjuvant treatment to decrease the risk of developing CKD in hepatitis C patients.

## 1. Introduction

Hepatitis is a serious issue facing the global health care community, with the hepatitis B virus (HBV) and hepatitis C virus (HCV), in particular, accounting for 96% of all hepatitis mortalities. As reported by World Health Organization (WHO) in 2015, an estimated 257 million people suffered from chronic hepatitis B (CHB) worldwide, while 71 million people suffered from chronic hepatitis C (CHC) [1].

According to two previous nationwide cohort studies investigating CHB and CHC patients in Taiwan, the risk of developing chronic kidney disease (CKD) was approximately 2.3-folds higher in the CHB cohort compared with the non-CHB cohort, while that risk was 1.66-fold higher in the CHC cohort than the non-CHC cohort [2, 3], indicating that chronic hepatitis patients have an elevated risk of developing CKD. Moreover, hepatitis patients associated with CKD will present enhanced obstacles to treatment, and an increased mortality rate, both of which further increase the economic burden placed on health care systems.

HBV infection not only affects liver function but also induces HBV-associated glomerulonephritis with several renal manifestations, particularly membranous nephropathy (MN). The HBV may interact with pre-existing host factors leading to a possible increase in morbidity and mortality [4]. HCV infection increases the risks of developing CKD and progression to end-stage renal failure (ESRF), associated with an elevated mortality rate observed in kidney dialysis and transplant recipients. Meanwhile, Solid et al. found that CHC patients with CKD G1-G5 and end-stage renal disease (ESRD) demonstrate a three-fold increased mortality rate than non-CKD patients. In a separate study of dialysis patients, the HCV cohort exhibited a higher adjusted hazard ratio (aHR) for mortality than the non-HCV cohort [5, 6].

Medical options for the treatment of CHB generally range from the injected form of interferon-*α* to the oral forms of nucleoside analogs (NAs). NAs used in the treatment of CHB patients are generally considered effective and convenient due to their oral administration and aside from telbivudine, exhibit minimal negative effects on renal function [7, 8]. However, with progression to CKD, interferon-*α* is not suitable due to poor tolerance, injection risks, and low effectiveness. Some NAs must be adjusted according to renal function (creatinine clearance <50 mL/min), and nephrotoxicity with adefovir and tenofovir treatments must be considered [8, 9].

For the decade prior to the development of direct-acting antivirals (DAAs), interferons (IFN), or Peginterferons (PEG-IFN) combined with ribavirin were the primary therapeutic modalities for HCV. Early DAAs, prevalent from 2011 to 2013, still required combination with PEG-IFN [10]. Both IFN and ribavirin are metabolized through the kidneys, thus increasing the complexity of treating HCV patients with CKD, and demonstrate poor effectiveness when associated with ESRF and hemodialysis [11].

Due to enhanced treatment complexity, the management of hepatitis patients with CKD will inevitably result in higher medical expenses incurred by health care systems. A U.S. study reported that the health care utilization rate was higher for CHB-CKD patients, with financial expenditures 3 to 5 times higher compared to non-CKD CHB patients [12]. Furthermore, a retrospective cohort study of 35,965 HCV patients concluded that HCV patients with CKD had higher health care utilization rates and medical costs [5]. A separate retrospective cohort study of 3,438 HCV patients receiving oral DAAs revealed that those with CKD also incurred higher health care costs compared with non-CKD HCV patients [13].

The development of new NAs and DAAs has significantly increased the response rate for both HBV and HCV patients with CKD; however, there are limitations primarily related to their high expenses. As such, patients in Taiwan often seek complementary therapy from TCM, which is widely covered by the National Health Insurance system. Previous studies have revealed that nearly 37% of patients with HBV and 66% of patients with HCV utilized Chinese herbal medicine (CHM) as an adjuvant therapy in Taiwan [14, 15]. In addition, a recent study has suggested that CHM could be considered an effective adjunct to prolong the lifespan of patients with CKD [16]. However, despite the increased popularity of CHM as complimentary therapy, it remains unclear whether CHM can prevent the development of CKD in patients suffering from CHB and CHC.

Thus, the aim of the present study is to investigate CKD incidence among CHB and CHC patients between a CHM and non-CHM cohort by analyzing one million cases recorded in the Longitudinal Health Insurance Database (LHID2010) of the Taiwan NHIRD. Furthermore, we have collected and analyzed the associated CHM prescription patterns to provide prescription guidance for clinical practitioners and direction for future research.

## 2. Materials and Methods

### 2.1. Data Source

The Taiwan National Health Insurance (NHI), covering more than 99% of the population, set up the National Health Insurance Research Database (NHIRD) in 1995. The database contains outpatient treatment records, various hospitalization data, prescribed medications, and other medical data for each case; importantly, the database records of outpatient visits also include TCM outpatient service.

For this study, data from the Longitudinal Health Insurance Database (LHID) were used, which randomly selects one million subjects from the NHIRD, containing cases with age and gender distributions similar to the original database. To ensure the privacy of individuals, identification numbers are encrypted before the database is released. The diagnoses are coded according to the International Classification of Disease, Ninth Revision, Clinical Modification (ICD-9-CM). The Research Ethics Committee of China Medical University and Hospital in Taiwan approved this study (CMUH-104-REC2-115-R3).

### 2.2. Study Population

The population included in this study were patients newly diagnosed with hepatitis (HBV, ICD-9-CM: 070.22, 070.23, 070.32, 070.33, and V02.61; HCV, ICD-9-CM: 070.41, 070.44, 070.51, 070.54, and V02.62) during the period of 2000 to 2010. The CHM users were defined as patients with at least 30 days prescription of CHM, and the first date of CHM prescription was set as index date during 2000 to 2012. We then categorized the hepatitis patients into two cohorts, with or without use of CHM after hepatitis diagnosis. All subjects selected were over 18 years of age and followed up to CKD occurrence, The expired date was set as the patients was dead, withdrawn by NHIRD or out of Dec 31, 2013.

### 2.3. Primary Outcome

The primary outcome of this study was patients newly diagnosed with CKD (ICD-9-CM: 250.4, 274.1, 283.11, 403.1, 404.2, 404.3, 440.1, 442.1, 447.3, 572.4, 580–588, 642.1, and 646.2) after hepatitis diagnosis. As the risk of outcome may be influenced by comorbidities and the usage of the medicine, histories of hypertension (ICD-9-CM: 401–405), diabetes mellitus (ICD-9-CM: 250), coronary artery disease (ICD-9-CM: 410–414), hyperlipidemia (ICD-9-CM: 272.0, 272.1, 272.2, 272.4), cirrhosis (ICD-9-CM: 571.2, 571.5, 571.6), congestive heart failure (ICD-9-CM: 428.0), osteoporosis (ICD-9-CM: 733.0, 733.1), arrhythmia (ICD-9-CM: 427.89, 427.9), peripheral vascular disease (ICD-9-CM: 443.89, 443.9), HBV drugs (lamivudine, adefovir, entecavir, telbivudine, and tenofovir), HCV drugs (peginterferon), CKD drugs (dipyridamole, pentoxifylline, acei, arb, ccb, acetylcysteine, allopurinol, febuxostat, benzbromarone, vitamin D), DM drugs (metformin, DPP4, sulfonylureas, TZD, other anti-DM drugs) and statin were included as covariates; meanwhile, age, gender, and urbanization were demographic factors adjusted in this study.

### 2.4. Matching

For each newly diagnosed chronic hepatitis patient with CHM treatment, we used propensity score matching at 1 : 1 to select the corresponding matched non-CHM users. Propensity score calculated the probability by using the logistic regression model and the variables including age, gender, urbanization, comorbidities, and medications mentioned above.

### 2.5. Statistical Analyses

To compare the differences in demographic factors, comorbidities, and correlated drugs between the CHM and non-CHM cohorts, chi-squared test and *t*-test were used for category and continuous variables, respectively. We then calculated the risk of CKD between CHM and non-CHM cohorts. The crude and adjusted Cox proportional hazard models were demonstrated by hazard ratios (HRs) and aHRs with 95% confidence intervals (CIs). The Kaplan–Meier method was applied to measure cumulative incidence curves, and the log-rank test was used to evaluate the difference between the two curves and plotted by R studio (3.5.2). The network plot analysis by the open source free software, NodeXL (http://nodexl.codeplex.com/), revealed the associations of any two herbs and CHM formulas. The statistical analysis ran with type I error *α* = 0.05 using the statistical software package, SAS, version 9.4 (SAS Institute, Inc., Cary, NC).

## 3. Results

### 3.1. Demographic Characteristics

**Figure 1:**
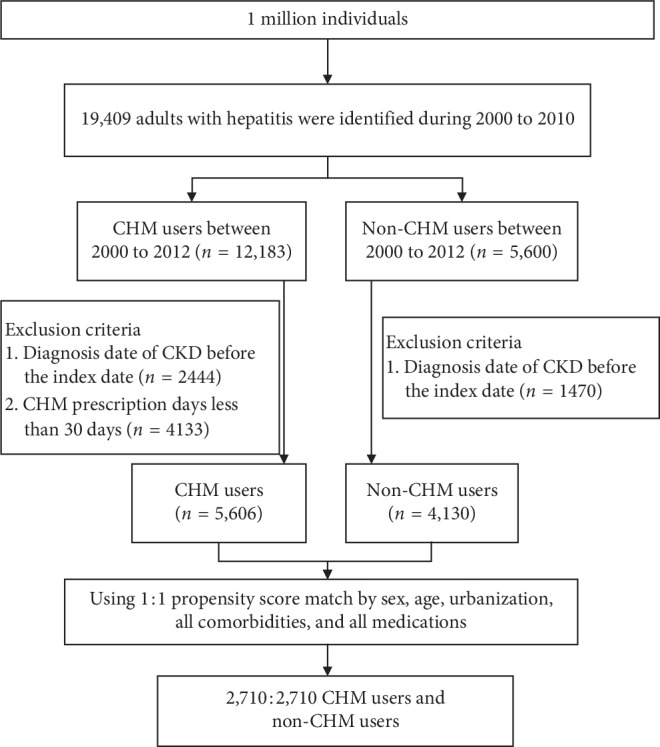
Flow chart of study cases (Chinese herbal medicine, CHM) from the Longitudinal Health Insurance Database (LHID) in Taiwan, from years 2000 to 2010.

Of the one million individual cases recorded in the LHID, 19,409 adults diagnosed with hepatitis between 2000 and 2010 were identified. After exclusion criteria and determination of CHM use, those patients were classified into two groups, CHM and non-CHM users. With propensity score matching by sex, age, urbanization, comorbidities, and medications, the CHM and non-CHM cohorts each consisted of 2,710 subjects (Figure 1). The study population of CHM users and non-CHM users, shown in Table 1, were approximately 60% male with a mean age of 53.2 years. In terms of medication use, more than 50% of hepatitis patients were treated for CKD, followed by diabetes mellitus (DM). The only covariate which showed a significant difference between the two groups was age (*p* value = 0.01).

### 3.2. The Risk Factors and Cumulative Incidence of CKD in Patients with Hepatitis

As shown in Table 2, patients diagnosed with CKD after hepatitis diagnosis were filtered out, and Cox model was used to adjust the HR of CKD incidence. The results of the study revealed that 318 hepatitis patients in the non-CHM cohort, and 452 patients in the CHM cohort developed CKD. After adjustment for gender, age, urbanization, comorbidities, and medications, patients in the CHM cohort exhibited a lower cumulative incidence of CKD (aHR = 0.69, *p* value ≤0.001, 95% CI = 0.59–0.8) in comparison with the non-CHM cohort. Of note, males had a higher risk of developing CKD, while age was also found to be highly correlated to increased incidence of CKD.

With regards to comorbidities, patients with heart failure (aHR = 1.61, 95% CI = 1.23–2.11), diabetes mellitus (aHR = 1.55, 95% CI = 1.31–1.84), liver cirrhosis (aHR = 1.54, 95% CI = 1.29–1.83), and hypertension (aHR = 1.36, 95% CI = 1.14–1.62) demonstrated significant differences (*p* value <0.001) in the development of CKD. It is worth noting that only DM medication increased the risk of developing CKD among hepatitis patients (aHR = 1.41, 95% CI = 1.18–1.68, *p* value <0.001). HBV drugs had no significant effect to prevent development of CKD (*p* value = 0.15); however, HCV, statin, and CKD medications reduced the risk of developing CKD in patients with hepatitis.

Multivariable stratified analyses were applied to verify the association of hepatitis patients with CHM treatment to reduce CKD risk for those comorbidities. The results demonstrated statistical significance for hepatitis patients associated without or with these comorbidities including hypertension (aHR = 0.69, 95% CI = 0.54–0.88; aHR = 0.70, 95% CI = 0.58–0.85), diabetes (aHR = 0.73, 95% CI = 0.60–0.90; aHR = 0.67, 95% CI = 0.54–0.84), liver cirrhosis (aHR = 0.79, 95% CI = 0.67–0.94; aHR = 0.50, 95% CI = 0.36–0.68), and heart failure (aHR = 0.73, 95% CI = 0.62–0.85; aHR = 0.51, 95% CI = 0.31–0.86) between these two cohorts. These results indicate that CHM significantly decreases the CKD risk in hepatitis patients associated with or without comorbidities including hypertension, diabetes mellitus, liver cirrhosis, and heart failure (Supplementary [Supplementary-material supplementary-material-1]).

**Figure 2:**
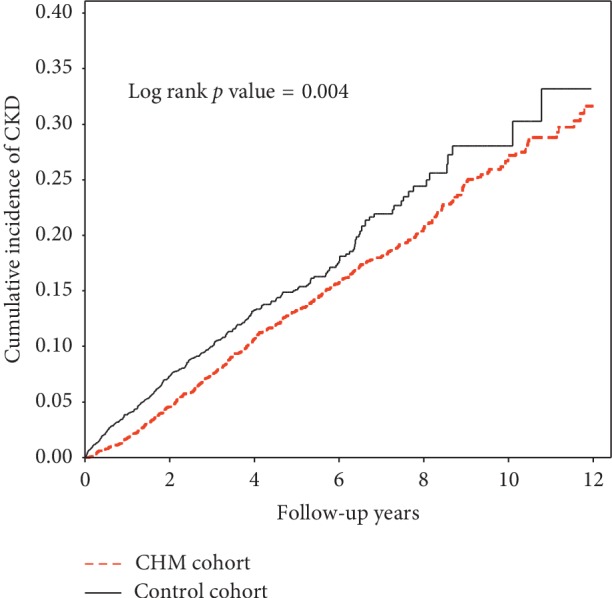
Cumulative incidence of CKD of both CHM and non-CHM cohorts in patients with hepatitis, by Kaplan–Meier analysis.

Additionally, the cumulative incidence of CKD, as calculated by the Kaplan–Meier method, revealed a significant difference (*p* value <0.05) between the CHM cohort and the non-CHM cohort in patients with hepatitis (Figure 2), indicating that the CHM cohort indeed had a lower cumulative incidence of CKD compared with the non-CHM cohort.

In order to clarify whether a specific type of hepatitis benefitted more from CHM, we further analyzed the HR of HBV, HCV, and HBV/HCV coinfection patients, as shown in Table 3. Notably, we found that CHM had reduced the risk of CKD incidence in the HCV (*p* < 0.001) and HBV/HCV coinfection (*p* < 0.05) subgroups; meanwhile, the aHR of the HBV subgroup was 0.81, demonstrating no statistical significance (*p* > 0.05). Furthermore, CHM users among hepatitis patients had longer follow-up periods represented by higher person years (15533 PY) compared with those in non-CHM cohort (9069 PY). This finding indicates that CHM may provide more protective effects to the renal function of CHC patients in particular, thereby reducing the risk of developing CKD. Considering the combination treatment of CHM and western medicine in HBV, HCV, and HBV/HCV coinfection patients (Table 4), HCV patients with western medicine (HCV drugs) treatment only (aHR = 0.66, 95% CI = 0.53–0.81), and patients receiving both CHM and HCV western medicine treatment (aHR = 0.40, 95% CI = 0.21–0.76) had significantly decreased risk of developing CKD. This indicates that CHM may indeed alleviate the risk of CKD in HCV patients.

In this study, the CHM users were defined as with at least 30 days prescription of CHM after hepatitis diagnoses. In Table 5, we stratified the prescription days of CHM users into 30 to 60 days, 60 to 90 days, and more than 90 days. We discovered that CHM users with at least 90 days of CHM consumption had significant lower risk of developing CKD compared with non-CHM users (aHR = 0.58, 95% CI = 0.49–0.69). Besides, there was a trend effect with lower CKD risk in parallel with CHM consumption periods (*p* for trend <0.001).

### 3.3. Top Ten Single Herbs and Formulas

**Figure 3:**
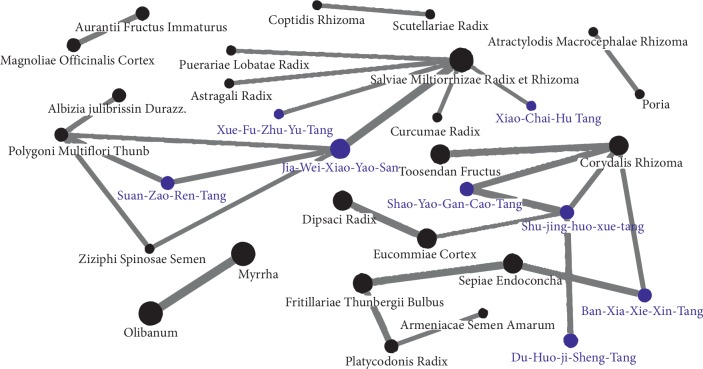
Network analysis of the 30 most frequently prescribed herbs and formulas for patients with hepatitis. The spot size indicates the frequency of Chinese herbal product prescription, and the line width indicates the combination frequency between two Chinese herbal products.

The top ten single herbs and formulas prescribed by TCM practitioners for the treatment of hepatitis are listed in Table 6. The three most frequently prescribed single herbs were *Salvia miltiorrhiza*, *Corydalis yanhusuo*, and *Radix et Rhizoma Rhei*. The average days of prescription for single herbs ranged from 7.1 to 9.2 days. The three most frequently prescribed formulas were Jia-Wei-Xiao-Yao-San (JWXYS), Xiao-Chai-Hu Tang (XCHT), and Shu-Jing-Huo-Xue-Tan (SJHXT). The average days of prescription for formulas ranged from 7 to 9 days, very similar to single herbs. To clarify the association between CHM and CKD, the top ten single herbs and formulas for each hazard ratio of developing CKD are presented in Table 7. Except *Astragali Radix*, Ban-Xia-Xie-Xin-Tang, and Long-Dan-Xie-Gan-Tang, the single herbs and formulas led to significant lower risk of developing CKD (all of *p* < 0.05). A network analysis of the thirty most frequently prescribed single herbs and formula combinations is shown in Figure 3, revealing JWXYS and *SM*, as well as *Olibanum* and *Myrrha* as the most frequent combinations.

## 4. Discussion

This is the first large-scale nationwide cohort study exploring the association between CHM therapy and incidence of CKD in hepatitis patients. The results of the study reveal that CHM, as an adjuvant therapy for patients with hepatitis, indeed offered protective effects and prevented the development of CKD, particularly in the HCV subtype.

There are several mechanisms at play in hepatitis patients. In particular, HBeAg is prone to combine with host antibodies, forming an immune-complex which affects renal function and induces the inflammatory process resulting in secondary MN, the most common renal manifestation in patients with HBV [17]. Approximately 40% to 50% of MN in adults results in nephrotic syndrome, eventually progressing to ESRD within a period of 6 to 13 years [18]. Thus, HBeAg seroconversion may be critical in the prevention of renal damage. As reported by Chen et al., HBV patients without NAs treatment will more easily develop ESRD [19]. Meanwhile, HCV can induce renal dysfunction via direct infection of kidney tissue, lymphotropism, and cryoglobulinemia [10]. HCV directly infects kidney tissue via CD81 expression on its surface to induce cell apoptosis [20]. Type II mixed cryoglobulinemia, originating from IgG-IgM complexes, is the primary manifestation in HCV patients, predominantly inducing type I membranoproliferative glomerulonephritis (MPGN) as a result of cryoglobulinemic vasculitis [10, 21]. The prevalence of type II mixed cryoglobulinemia-dependent MPGN in CHC patients is approximately 30% [22] and is considered the most common cause of nephritic or nephrotic syndrome [23]. In a large-scale cohort study (*n* = 56,448), CHC patients demonstrated an approximately 2-fold and 17-fold risk of developing MPGN and cryoglobulinemia, respectively [24]. Thus, antiviral therapy may be a superior option to protect kidneys by preventing viral proliferation and synthesis of immune-complexes, decreasing immune-complex and cryoglobulin formations, as well as preventing inflammation and vasculitis.

The present study explores the risk factors associated with CKD development in patients with hepatitis, such as male gender, age over 60, hypertension, DM, liver cirrhosis, and heart failure. Moreover, while HCV, CKD, and statin medications can decrease the risk of developing CKD, and DM medications, by contrast, can increase the risk. As previously reported, male gender and age are the primary risk factors associated with the development of MN, while the average onset of MN was in patients' 50s and 60s of age, compatible with the findings of this study [18]. Meanwhile, hypertension exerts negative effects on the kidneys as it leads to increased intraglomerular capillary pressure, podocyte dysfunction, abnormal permeability, and eventual proteinuria. This mechanism can be prevented via angiotensin-converting enzyme inhibitors (ACEIs), and angiotensin receptor blockers (ARBs) [25].

The development of MN or MPGN caused by hepatitis viral infection will lead to nephrotic syndrome, associated with proteinuria. The protein loss results in a decrease of oncotic pressure, and hypoproteinemia will be compensated for by liver genesis lipoprotein; consequently, excess production of low density lipoprotein causes hypercholesteremia. However, hypocholesteremia has been observed in HCV patients due to upregulated lipid biosynthesis and impaired lipid degradation, which leads to intracellular lipid accumulation and facilitates the formation of steatosis and fibrosis [26]. This phenomenon will induce hypoproteinemia and affect the cardiovascular system as a result of hypertension and/or congestive heart failure, becoming risk factors of CKD development in patients with hepatitis. Statin has been reported to control hyperlipidemia, exert powerful anti-HCV effects, and thus reduce the risk of cirrhosis in hepatitis patients [27].

Additionally, DM is a common extrahepatic manifestation associated with HCV infection [22]. The HCV core protein decreases the expression of insulin receptor substrate proteins (IRS) 1 and 2 and causes abnormal insulin sensitivity and beta-cell dysfunction. As such, it activates insulin resistance and hyperinsulinemia, consequently inducing HCV-related diabetic nephropathy [20]. Many DM drug metabolites are eliminated via the kidneys, such as biguanide, sulphonylureas, dipeptidylpeptidase-4 inhibitors, and SGLT-2 inhibitors, possibly burdening renal function [28].

Based on the top 10 most frequently prescribed formulas and single herbs, CHM may prevent CKD incidence among hepatitis patients via directly inhibiting viral expression, preventing liver and kidney damage, treating the comorbidities, and alleviating the side effects induced by interferon. HBV and/or HCV infection will activate the inflammatory process, leading to fibrosis and cirrhosis. According to TCM theory, these phenomena are primarily patterned as hepatobiliary damp-heat. Among the top ten herbs and formulas, XCHT, *Radix et Rhizoma Rhei*, *Radix astragali*, and *Scutellaria radix* exert anti-HBV activity, while XCHT has exhibited anti-HCV activity in a phase II clinical trial [29].

The effects of cryoglobulinemia and immune-complexes cause renal function decline. These, according to TCM theory, can be considered consistent with Qi stagnation and blood stasis. Several CHM herbs and formulas may offer protective effects against both liver and renal function decline. In particular, TCM theory suggests that JWXYS and Xue-Fu-Zhu-Yu-Tang (XFZYT) may serve to soothe liver Qi and relieve blood stasis. Studies have reported that JWXYS may improve the survival rate in HBV patients when combined with lamivudine treatment [30] and decrease the mortality rate and risk of liver cirrhosis in HCV patients [31]. Furthermore, JWXYS has been shown to elevate serum albumin levels and prevent liver fibrosis [32]. Meanwhile, TCM theory suggests that *SM*, the most commonly prescribed herb identified in this study, can invigorate blood, dispel blood stasis, and cool blood heat. Furthermore, it has been reported that *SM* prevents immune-mediated liver injury and liver fibrosis through modulating the NF-κB and IFN-γ/STAT1 signaling pathways as well as enhancing NK cell activity [33]. Of note, a recent study revealed renal fibrosis resulting from MN and diabetic nephropathy via the dysregulation of the TGF-*β*/Smad pathway [34]. This pathway can be regulated by *SM* and *Scutellaria radix* to protect renal function from deterioration [35].

On the aspect of treating the comorbidities, XFZYT has not only antiangiogenesis effect that can prevent liver fibrosis [36] but also lowers the blood pressure [37]. *Pueraria lobate* exerts anti-inflammation effects through regulating the IKKb/IRS-1 pathway and improving endothelial insulin resistance. When combined with *SM*, the synergistic effects of antihypertension and antiatherosclerosis may effectively reduce the risk of CKD [38, 39]. This correlation was further observed in the network analysis herein. SM alone can also prevent the development of DM nephropathy via suppressing TGF-*β*1 and improving renal microcirculation [40].

Other herbal formulas without direct hepatic and renal protective activities may help deal with the interferon side effects. Some HCV patients undergoing pegylated interferon/ribavirin therapy will seek CHM to alleviate side effects and improve quality of life [41]. The common side effects of PEG-IFN include flu-like symptoms (headache, myalgia, and fatigue); digestive discomforts (nausea, loss of appetite, and diarrhea); and neuropsychiatric disorders (insomnia and depression) [42]. Shu-Jing-Huo-Xue-Tang and Shao-Yao-Gan-Cao-Tang have been reported to increase blood circulation and decrease muscle cramps to relieve myalgia [43, 44]. Ban-Xia-Xie-Xin-Tang possesses prokinetic activity and motility regulation to alleviate anorexia and diarrhea [45]. JWXYS and Suan-Zao-Ren-Tang improve insomnia and depression [46]. The other known pharmacological mechanisms of the top ten single herbs and formulas are illustrated in Supplementary Tables [Supplementary-material supplementary-material-1] and [Supplementary-material supplementary-material-1].

Although the new generation of NAs and DAAs provides hepatitis patients with effective treatment options, high costs may restrict their popularity and availability, particularly in developing countries. By contrast, CHM treatments offer more cost-effective management strategies. This study demonstrates that CHM provided protective effects against development of CKD for HCV patients. Therefore, the use of CHM as an adjuvant therapy strategy warrants consideration by medical practitioners.

Inevitably, the analysis of data extracted from the NHIRD has limitations. First, laboratory data, images, pathology reports, and patient lifestyle details are not recorded in the database. Second, the diagnostic records were based on ICD-9, which lacks data regarding CKD severity; ICD-10 provides more information (ICD-10 code: N181–N189) regarding the severity of CKD. Third, some CKD risk factors, such as proteinuria, smoking, herbal supplement therapy, intravenous drug use, and human immunodeficiency virus infection were not included in this study. Fourth, we did not exclude the influence from hepatitis D virus infection among CHB groups. Furthermore, patients' CHM compliance cannot be shown by NHIRD.

## 5. Conclusions

The database analysis of this study reveals that CHM as an adjunctive therapy decreased incidence of CKD in Taiwanese patients with hepatitis C. These findings suggest that future research into complementary and alternative medicine treatments for patients with hepatitis C is warranted. However, future prospective cohort research is recommended to ascertain and elucidate the specific CHM mechanisms associated with CKD prevention in hepatitis patients.

## Figures and Tables

**Table 1:** Characteristics of hepatitis patients with and without CHM.

Characteristic	CHM		*p* value
	No *N* = 2710	Yes *N* = 2710	
	*n* (%)	*n* (%)	
*Gender^∗^*			0.09
Female	1094 (40.4)	1033 (38.1)	
Male	1616 (59.6)	1677 (61.9)	

*Age, year* ^*†*^			0.01
18–44	926 (34.2)	828 (30.6)	
45–59	873 (32.2)	972 (35.9)	
≥60	911 (33.6)	910 (33.6)	
Mean (SD)	53.2 (16.1)	53.2 (14.58)	0.97

*Urbanization^∗^*			0.29
1 (highest)	677 (25)	646 (23.8)	
2	792 (29.2)	835 (30.8)	
3	409 (15.1)	437 (16.1)	
4 (lowest)	832 (30.7)	792 (29.2)	

*Comorbidity^∗^*			
Hypertension	1124 (41.5)	1117 (41.2)	0.85
Diabetes mellitus	769 (28.4)	751 (27.7)	0.59
Coronary artery disease	734 (27.1)	723 (26.7)	0.74
Hyperlipidemia	789 (29.1)	776 (28.6)	0.7
Liver cirrhosis	474 (17.5)	457 (16.9)	0.54
Heart failure	123 (4.5)	113 (4.2)	0.51
Osteoporosis	367 (13.5)	359 (13.2)	0.75
Arrhythmia	310 (11.4)	289 (10.7)	0.36
PVD	66 (2.4)	65 (2.4)	0.93

*Medication^∗^*			
HBV drug	121 (4.5)	123 (4.5)	0.9
HCV drug	177 (6.5)	165 (6.1)	0.5
CKD drug	1512 (55.8)	1491 (55)	0.57
DM drug	467 (17.2)	474 (17.5)	0.8
Statin	320 (11.8)	315 (11.6)	0.83

^∗^Chi-squared test; ^†^*t*-test. Abbreviations: CHM, Chinese herbal medicine; SD, standard deviation; PVD, peripheral vascular disease; HBV, hepatitis B virus; hepatitis C virus; CKD, chronic kidney disease; DM, diabetes mellitus.

**Table 2:** Risk factors of CKD among hepatitis patients.

Variable	CKD	Crude			Adjusted^§^		
	Event no.	HR	95% CI	*p* value	HR	95% CI	*p* value
*CHM*							
No	318	1	(Reference)		1	(Reference)	
Yes	452	0.8	(0.69–0.93)	0.004	0.69	(0.59–0.8)	<0.001

*Gender*							
Female	312	1	(Reference)		1	(Reference)	
Male	458	0.9	(0.78–1.04)	0.14	1.26	(1.08–1.48)	0.003

*Age, year*							
18–44	108	1	(Reference)		1	(Reference)	
45–59	240	2.31	(1.84–2.9)	<0.001	2.12	(1.67–2.69)	<0.001
≥60	422	4.81	(3.89–5.94)	<0.001	3.59	(2.78–4.64)	<0.001

*Urbanization*							
1 (highest)	164	1	(Reference)		1	(Reference)	
2	227	1.1	(0.9–1.35)	0.35	1.12	(0.91–1.37)	0.29
3	109	1.02	(0.8–1.3)	0.89	0.98	(0.77–1.25)	0.85
4 (lowest)	270	1.39	(1.14–1.68)	<0.001	1.14	(0.93–1.38)	0.21

*Comorbidity*							
Hypertension	460	2.49	(2.16–2.88)	<0.001	1.36	(1.14–1.62)	<0.001
Diabetes mellitus	360	2.59	(2.25–2.99)	<0.001	1.55	(1.31–1.84)	<0.001
Coronary artery disease	303	2.05	(1.78–2.37)	<0.001	1.12	(0.95–1.33)	0.18
Hyperlipidemia	286	1.59	(1.37–1.84)	<0.001	1.13	(0.96–1.32)	0.14
Liver cirrhosis	180	2.02	(1.71–2.39)	<0.001	1.54	(1.29–1.83)	<0.001
Heart failure	65	2.84	(2.2–3.66)	<0.001	1.61	(1.23–2.11)	<0.001
Osteoporosis	147	1.74	(1.45–2.08)	<0.001	0.99	(0.82–1.21)	0.96
Arrhythmia	112	1.57	(1.29–1.92)	<0.001	0.97	(0.78–1.2)	0.78
PVD	23	1.49	(0.98–2.26)	0.06	0.96	(0.63–1.46)	0.85

*Medication*							
HBV drug	22	0.68	(0.45–1.04)	0.08	0.73	(0.47–1.12)	0.15
HCV drug	29	0.44	(0.3–0.63)	<0.001	0.45	(0.31–0.65)	<0.001
CKD drug	557	1.86	(1.58–2.17)	<0.001	0.78	(0.65–0.95)	0.01
DM drug	253	2.24	(1.93–2.6)	<0.001	1.41	(1.18–1.68)	<0.001
Statin	112	1.09	(0.89–1.33)	0.4	0.7	(0.56–0.86)	<0.001

CHM, Chinese herbal medicine; SD, standard deviation; PVD, peripheral vascular disease; HBV, hepatitis B virus; hepatitis C virus; CKD, chronic kidney disease; DM, diabetes mellitus; HR, hazard ratio; CI, confidence interval. ^§^Cox model adjusted with gender, age, urbanization, comorbidity and medication.

**Table 3:** The CHM effect on CKD in subgroups.

Subgroup	Non-CHM users			CHM users			Crude HR (95%)	Adjusted HR (95%)^§^
	Event no.	PY	IR	Event no.	PY	IR		
HBV	111	4216	2.63	134	5562	2.41	0.89(0.69–1.15)	0.81(0.62–1.05)
HCV	153	3384	4.52	281	8637	3.25	0.72 (0.59–0.88)^*∗∗*^	0.65 (0.53–0.8)^*∗∗∗*^
HBV and HCV	54	1469	3.68	37	1334	2.77	0.75 (0.49–1.14)	0.6 (0.39–0.92)^*∗*^

HBV, hepatitis B virus; hepatitis C virus; PY, person year; IR, incidence rate/per 100 person-years; HR, hazard ratio; CI, confidence interval. ^§^Cox model adjusted with gender, age, urbanization, comorbidity, and medication. ^*∗*^*p* < 0.05; ^*∗∗*^*p* < 0.01; ^*∗∗∗*^*p* < 0.001.

**Table 4:** Cox proportional hazard regression analysis for the risk of CKD for HBV and HCV medication.

Variable		Number	CKD event	Person years	IR	Crude HR (95% CI)	Adjusted HR (95% CI)
*HBV patients*							
CHM	HBV drug						
No	No	1169	107	4054	2.64	Ref.	Ref.
No	Yes	1080	134	5431	2.47	0.91 (0.71–1.18)	0.84 (0.65–1.10)
Yes	No	36	4	163	2.45	0.92 (0.34–2.50)	0.98 (0.36–2.67)
Yes	Yes	23	0	132	0.00	—	—

*HCV patients*							
CHM	HCV drug						
No	No	966	148	3100	4.77	Ref.	Ref.
No	Yes	1172	271	8060	3.36	0.70 (0.57–0.86)^*∗∗∗*^	0.66 (0.53–0.81)^*∗∗*^
Yes	No	58	5	284	1.76	0.37 (0.15–0.89)^*∗*^	0.52 (0.21–1.28)
Yes	Yes	78	10	577	1.73	0.36 (0.19–0.68)^*∗∗*^	0.40 (0.21–0.76)^*∗∗*^

*HCV and HBV patients*							
CHM	Both						
No	No	420	48	1258	3.82	Ref.	Ref.
No	Yes	325	35	1204	2.91	0.76 (0.49–1.18)	0.69 (0.44–1.09)
Yes	No	48	4	154	2.59	0.68 (0.25–1.89)	0.69 (0.24–1.97)
Yes	Yes	20	2	82	2.43	0.63 (0.15–2.57)	0.81 (0.19–3.39)

IR, incidence rates, HR, hazard ratio; CI, confidence interval. Adjusted HR: adjusted for sex, age, and all comorbidites in Cox proportional hazards regression. ^*∗*^*p* value <0.05; ^*∗∗*^*p* value <0.01; ^*∗∗∗*^*p* value <0.001.

**Table 5:** Hazard Ratios and 95% confidence intervals of CKD risk associated with cumulative use day of TCM among hepatitis patients.

	Event no. (*n* = 770)	Person year	IR	Hazard ratio (95% CI)	
				Crude	Adjusted^†^
Non-TCM users	318	9069	3.51	1.00 (ref.)	1.00 (ref.)
Chinese herb users					
30–60 days	144	4045	3.56	0.99 (0.81–1.20)	0.88 (0.72–1.07)
60–90 days	74	2253	3.28	0.91 (0.71–1.17)	0.82 (0.64–1.06)
>90 days	234	9236	2.53	0.70 (0.58–0.83)^*∗∗∗*^	0.58 (0.49–0.69)^*∗∗∗*^
*p* for trend					*p* < 0.001

Crude HR^*∗*^ represented relative hazard ratio; adjusted HR^†^ represented adjusted hazard ratio: mutually adjusted with gender, age, urbanization, comorbidity, and medication in Cox proportional hazard regression. ^*∗*^*p* < 0.05, ^*∗∗*^*p* < 0.01, ^*∗∗∗*^*p* < 0.001.

**Table 6:** The top ten single herbs and formulas.

	Frequency	Total prescription days	Total prescription dose	Average dose	Average days
*Single herb*					
*Salviae Miltiorrhizae*	18286	167551	421569.9	2.5	9.2
*Corydalis Rhizoma*	15145	107063	291003.8	2.7	7.1
*Rhei Radix et Rhizoma*	11676	91721	132202.1	1.4	7.9
*Scutellariae Radix*	11294	88082	233831.4	2.7	7.8
*Astragali Radix*	9310	82461	193854.9	2.4	8.9
*Fritillariae Thunbergii Bulbus*	11204	81940	216510.7	2.6	7.3
*Platycodonis Radix*	11343	76509	156414.6	2	6.7
*Puerariae Lobatae Radix*	10186	73121	166687.3	2.3	7.2
*Polygoni Multiflori Thunb*	8094	69468	208078.5	3	8.6
*Ziziphi Spinosae Semen*	8013	69061	312031.1	4.5	8.6

*Formula*					
Jia-Wei-Xiao-Yao-San	17136	153716	1408374	9.2	9
Xiao-Chai-Hu Tang	11782	93309	1008159	10.8	7.9
Shu-jing-huo-xue-tang	12959	90749	899737.6	9.9	7
Ban-Xia-Xie-Xin-Tang	11630	87996	942883.3	10.7	7.6
Shao-Yao-Gan-Cao-Tang	10471	70062	585790.8	8.4	6.7
Long-Dan-Xie-Gan-Tang	8580	69280	769180.1	11.1	8.1
Suan-Zao-Ren-Tang	9241	69113	760566.7	11	7.5
Xue-Fu-Zhu-Yu-Tang	8577	67528	528117.1	7.8	7.9
Xiang-Sha-Liu-Jun-Zi-Tang	8040	66951	499931.6	7.5	8.3
Ping-Wei-San	8777	65916	522327.2	7.9	7.5

**Table 7:** Hazard ratios and 95% confidence intervals of mortality risk associated with herbal formulas among hepatitis patients.

TCM prescription	CKD	Hazard ratio (95% CI)	
	No. of events	Crude^*∗*^	Adjusted^†^
Non-TCM user	318	Ref.	Ref.
*Single herb*			
*Salviae Miltiorrhizae*	201	0.92 (0.77–1.11)	0.73 (0.61–0.89)^*∗∗*^
*Corydalis Rhizoma*	194	0.87 (0.72–1.05)	0.74 (0.61–0.89)^*∗∗*^
*Rhei Radix et Rhizoma*	108	0.95 (0.76–1.18)	0.75 (0.60–0.95)^*∗*^
*Scutellariae Radix*	145	0.76 (0.62–0.94)^*∗∗*^	0.72 (0.58–0.89)^*∗∗*^
*Astragali Radix*	127	1.00 (0.81–1.24)	0.83 (0.67–1.03)
*Fritillariae Thunbergii Bulbus*	152	0.84 (0.69–1.03)	0.74 (0.60–0.91)^*∗∗*^
*Platycodonis Radix*	153	0.95 (0.78–1.15)	0.81 (0.66–0.99)^*∗*^
*Puerariae Lobatae Radix*	143	0.82 (0.67–1.00)	0.71 (0.58–0.88)^*∗∗*^
*Polygoni Multiflori Thunb*	93	0.80 (0.63–1.01)	0.69 (0.54–0.88)^*∗∗*^
*Ziziphi Spinosae Semen*	96	0.86 (0.68–1.08)	0.76 (0.59–0.96)^*∗*^

*Formula*			
Jia-Wei-Xiao-Yao-San	150	0.85 (0.69–1.03)	0.80 (0.65–0.99)^*∗*^
Xiao-Chai-Hu Tang	165	0.87 (0.72–1.06)	0.74 (0.60–0.90)^*∗*^
Shu-jing-huo-xue-tang	196	0.91 (0.76–1.09)	0.75 (0.62–0.91)^*∗∗*^
Ban-Xia-Xie-Xin-Tang	145	0.96 (0.78–1.17)	0.82 (0.66–1.01)
Shao-Yao-Gan-Cao-Tang	162	0.80 (0.66–0.98)^*∗*^	0.69 (0.57–0.85)^*∗∗*^
Long-Dan-Xie-Gan-Tang	123	0.96 (0.77–1.19)	0.81 (0.65–1.01)
Suan-Zao-Ren-Tang	106	0.80 (0.64–1.01)	0.72 (0.57–0.91)^*∗∗*^
Xue-Fu-Zhu-Yu-Tang	144	0.91 (0.74–1.12)	0.75 (0.61–0.93)^*∗∗*^
Xiang-Sha-Liu-Jun-Zi-Tang	118	0.86 (0.69–1.06)	0.67 (0.54–0.84)^*∗∗*^
Ping-Wei-San	113	0.81 (0.65–1.01)	0.68 (0.54–0.85)^*∗∗*^

Crude HR^*∗*^ represented relative hazard ratio; adjusted HR^†^ represented adjusted hazard ratio: mutually adjusted for age group, gender, urbanization level, and cci score in Cox proportional hazard regression. ^*∗*^*p* < 0.05, ^*∗∗*^*p* < 0.01, ^*∗∗∗*^*p* < 0.001.

## Data Availability

The dataset is provided from Taiwan's National Health Research Institutes (http://nhird.nhri.org.tw).

## Abbreviations

aHR: Adjusted hazard ratios
CHB: Chronic hepatitis B
CHC: Chronic hepatitis C
CHM: Chinese herbal medicine
CI: Confidence interval
CKD: Chronic kidney disease
DAAs: Direct-acting antivirals
DM: Diabetes mellitus
ESRD: End-stage renal disease
ESRF: End-stage renal failure
HBV: Hepatitis B virus
HCV: Hepatitis C virus
ICD-9-CM: International Classification of Disease, Ninth Revision, Clinical Modification
IFN: Interferon
JWXYS: Jia-Wei-Xiao-Yao-San
MN: Membranous nephropathy
MPGN: Membranoproliferative glomerulonephritis
NAs: Nucleoside analogs
NHI: National Health Insurance
SM: *Salvia Miltiorrhiza*
TCM: Traditional chinese medicine
XCHT: Xiao-Chai-Hu Tang
XFZYT: Xue-Fu-Zhu-Yu-Tang.

## Supplementary Materials

Supplementary Materials Supplementary Table 1: incidence rates, hazard ratio, and confidence intervals of CKD in different stratifications correlated with comorbidities. Multivariable stratified analyses were applied to verify the association of hepatitis patients with CHM treatment to reduce CKD risk for those comorbidities. Supplementary Table 2: the hepatic and renal protective activities associated with experimental models of extracted ingredients of single herbs. The known pharmacological mechanisms of the top ten single herbs were associated with liver and renal protection by cell and/or animal experimental studies. Supplementary Table 3: the compositions as well as hepatic and renal protective activities associated with experimental models of top ten formulas. The compositions and hepatic protection of the top ten formulas were illustrated.
