# Supplementary material for: Chinese Herbal Medicine Ameliorated the Development of Chronic Kidney Disease in Patients with Chronic Hepatitis C: A Retrospective Population-Based Cohort Study
Source: Evid Based Complement Alternat Med. 2019 Nov 21;2019:5319456. doi: 10.1155/2019/5319456 (PMC6906860; doi:10.1155/2019/5319456)
Supplement: Supplementary Materials — Supplementary Table 1: incidence rates, hazard ratio, and confidence intervals of CKD in different stratifications correlated with comorbidities. Multivariable stratified analyses were applied to verify the association of hepatitis patients with CHM treatment to reduce CKD risk for those comorbidities. Supplementary Table 2: the hepatic and renal protective activities associated with experimental models of extracted ingredients of single herbs. The known pharmacological mechanisms of the top ten single herbs were associated with liver and renal protection by cell and/or animal experimental studies. Supplementary Table 3: the compositions as well as hepatic and renal protective activities associated with experimental models of top ten formulas. The compositions and hepatic protection of the top ten formulas were illustrated. [file 5319456.f1.docx]

**Supplementary Materials**

| **Supplementary Table 1** Incidence rates, hazard ratio and confidence intervals of CKD in different stratification of comorbidities including hypertension, diabetes mellitus, liver cirrhosis and heart failure | | | | | | | | | | |
| --- | --- | --- | --- | --- | --- | --- | --- | --- | --- | --- |
| **Variables** | **Control** | | |  | **Case** | | |  | **Case VS. Control** | |
|  | **N=2710** | | |  | **N=2710** | | |  | **Crude HR** | **Adjusted HR** |
|  | **CKD Event** | **Person years** | **IR** |  | **CKD Event** | **Person years** | **IR** |  | **(95% CI)** | **(95% CI)** |
| **Baseline comorbidity** |  |  |  |  |  |  |  |  |  |  |
| **Hypertension** |  |  |  |  |  |  |  |  |  |  |
| No | 124 | 5776 | 2.15 |  | 186 | 9600 | 1.94 |  | 0.86(0.68-1.08) | 0.69(0.54-0.88)** |
| Yes | 194 | 3294 | 5.89 |  | 266 | 5934 | 4.48 |  | 0.73(0.61-0.89)** | 0.70(0.58-0.85)** |
| **Diabetes mellitus** |  |  |  |  |  |  |  |  |  |  |
| No | 160 | 6810 | 2.35 |  | 250 | 11549 | 2.16 |  | 0.87(0.71-1.06) | 0.73(0.60-0.90)** |
| Yes | 158 | 2259 | 6.99 |  | 202 | 3985 | 5.07 |  | 0.72(0.58-0.89)** | 0.67(0.54-0.84)** |
| **Liver Cirrhosis** |  |  |  |  |  |  |  |  |  |  |
| No | 240 | 8196 | 2.93 |  | 350 | 13173 | 2.66 |  | 0.86(0.72-1.01) | 0.79(0.67-0.94)** |
| Yes | 78 | 874 | 8.93 |  | 102 | 2360 | 4.32 |  | 0.52(0.38-0.70)*** | 0.50(0.36-0.68)*** |
| **Heart failure** |  |  |  |  |  |  |  |  |  |  |
| No | 286 | 8823 | 3.24 |  | 419 | 14999 | 2.79 |  | 0.82(0.71-0.96)* | 0.73(0.62-0.85)*** |
| Yes | 32 | 247 | 12.97 |  | 33 | 535 | 6.17 |  | 0.51(0.31-0.84)** | 0.51(0.31-0.86)* |
| *Abbreviation: IR, incidence rates, HR, hazard ratio; CI, confidence interval | | | | | | | | | | |
| *Adjusted HR: adjusted for age, urbanization, comorbidities and all medications in Cox proportional hazards regression. | | | | | | | | | | |
| **p-*value <0.05 *; *p-*value <0.01 **; *p-*value<0.001*** | | | | | | | | | | |

**Supplementary Table 2** The hepatic and renal protective activities associated with experimental models of extracted ingredients of single herbs

| **Chinese material**  **medical name** | **Pin-yin name** | **Active ingredient or derivative** | **Possible pharmacological effects** |
| --- | --- | --- | --- |
| *Salviae Miltiorrhizae Radix* | Dan-Shen  (丹參) | 1.Protocatechuic aldehyde | 1. It inhibits hepatitis B virus replication [1]. |
|  |  | 2.Sodium tanshinone IIA sulfonate | 2. It protects mice from immune-mediated liver injury through modulating NF-κB and IFN-γ/STAT1 signaling pathways [2] and ameliorates ischemia/ reperfusion-induced kidney destruction in mice via targeting GSK3β [3]. |
|  |  | 3. Salvianolic acid A | 4.It reduces hepatic injury, interferon-gamma (IFN-γ), tumor necrosis factor-alpha (TNF-α), NF-κB and increases the expression of SIRT1 to prevent hypoxia damage [4]. |
|  |  | 4. Danshan extracts | 5. Its extracts improved the symptoms of glomerular and tubular atrophy [5]. |
|  |  |  | 6. Danshan enhances the activities of NK cell to reduce liver fibrosis [6].  7. Danshen can interact with intestinal bacteria from chronic renal failure rats [7]. |
| *Corydalis Rhizoma* | Yan-Hu-So  (延胡索) |  | n/a |
| *Rhei Radix et Rhizoma* | Da-Huang  (大黃) | 1.Bile acid metabolites | 1.It has hepatoprotective effect against α-naphthylisothiocyanate (ANIT)-induced liver injury [8]. |
|  |  | 2.Rhein | 2. It has antitumor and anti-inflammatory properties [9]. |
|  |  | 3. Chrysophanol 8-O-beta-D-glucoside | 3.It has strong anti-HBV activity on reduction extracellular HBV DNA of HepG2.2.15 cells [10]. |
| *Scutellariae Radix* | Huang-Qin  (黃芩) | 1. Baicalin | 1.It has antioxidant and anti-inflammatory effects toward liver and kidney disease via attenuating the activity of NF-kB and suppressing the expression of several inflammatory cytokines and chemokines [11].  2.It alleviates tubular-interstitial nephritis by down-regulating NF-κB and MAPK pathways [12] and inhibits pro-inflammatory responses and tubular apoptosis due to renal ischemia-reperfusion injury [13]. |
|  |  | 2.Woginin | 4.It has strong anti-HBV activity [10] and anti-fibrotic effect in renal tubular epithelial cells via TGF –β1/Smad3 signaling inhibition [14]. |
|  |  |  | 3. *Scutellariae Radix* acts as an anti-inflammatory agent by ameliorating oxidative stress in the liver [15].  4. *Scutellariae Radix* reduced inflammation, collagen accumulation ,and prevented liver fibrosis via inhibits stellate cell activation and proliferation by the down-regulation of PDGF-beta receptor [16]. |
| *Astragali Radix* | Huang-Qi  (黃耆) | 1.Astragaloside IV | 1.It has anti-fibrogenic effect to liver via oxidativestress-mediated p38 MAPK pathway [17] and inhibits renal tubule-interstitial fibrosis via blocking TGF-β/Smad signaling pathway [18]. |
| *Fritillariae Thunbergii Bulbus* | Zhe-Bei- Mu  (浙貝母) |  | n/a |
| *Platycodonis Radix* | Jie-Geng  (桔梗) | 1.Platycodin D | 1.Its anti-tumor ingredient induces apoptosis and triggers ERK- and JNK-mediated autophagy in human hepatocellular carcinoma BEL-7402 cells [19] and triggers autophagy in hepatocellular carcinoma HepG2 cells.via activation of extracellular signal-regulated kinase [20]. |
| *Puerariae Lobatae Radix* | Ge-Gen  (葛根) |  | Its constituents have anti-inflammatory and antioxidant activities [21]. |
| *Polygoni Multiflori Thunb*  (Part of application: stem) | Ye-Jiao -Teng  (夜交藤) |  | 1.It has slight anti-inflammatory activity ,but may contribute to its synergetic effect [22]. |
| *Ziziphi Spinosae Semen* | Suan-Zao -Ren  (酸棗仁) |  | 1.It possesses certain modulation effects on cytokines(IL-6 and IL-1β increased and TNF-α decreased), and the immuno-regulating function [23]. |

n/a :not available.

**Supplementary Table 3** The compositions as well as hepatic and renal protective activities associated with experimental models of top ten formulas.

| **Pin-yin name**  **Chinese name** | **Ingredients of herbal formula** | **Therapeutic actions and indications based on TCM theory** | **Possible pharmacological effects** |
| --- | --- | --- | --- |
| Jia-Wei-Xiao-Yao-San  加味逍遙散 | *Paeonia suffruricosa,  Gardenia jasminoides, Bupleurum chinense, Angelica sinensis,  Paeonia lactiflora,  Atractylodes macrocephala, Poriacocos,  Mentha haplocalyx,  Zingiber officinale and Glycyrrhiza uralensis* | 1. Disperses stagnated liver qi for relieving qi stagnation.  2. Clears heat and nourishing blood. | 1. Jia-Wei-Xiao-Yao-San displayed hepatoprotective effect on dimethylnitrosamine-induced chronic hepatitis and hepatic fibrosis in rats [24]. |
| Xiao-Chai-Hu Tang 小柴胡湯  **sho-saiko-to.** | *Bupleurum chinense,  Scutellaria baicalensis,  Pinellia ternate,  Zingiber officinale,  Panax ginseng,  ziziphus jujube and  Glycyrrhiza uralensis* | 1.Harmonizes shao yang stage disorders.  2.Harmonizes and tonifies the middle jiao.. | 1. Xiao-Chai-Hu Tang has anti-fibrosuppressant activity by inhibition of lipid peroxidation in hepatocytes and stellate cells in vivo [25] or activation of Nrf2 pathway [26]. 2. Xiao-Chai-Hu Tang can inhibit the production of HBV and decreased the expression of HBeAg [27]. 3.Xiao-Chai-Hu Tang may be useful in the prevention of HCV progress in addition, suppresses liver cancer development [28]. |
| Shu-jing-huo-xue-tang  疏經活血湯 | *Glycyrrhiza uralensis, Angelica sinensis., Paeonia lactiflora, Rehmannia glutinosa, Atractylodes lancea, Achyranthes bidentata, Citrus reticulata Blanco,*  *Prunus davidiana , Clematis chinensis, Ligusticum striatum, Stephania tetrandra, Notopterygium incisum, Saposhnikovia divaricata, Angelica dahurica, Gentiana scabra, Poria cocos, and  Zingiber officinale* | 1. Relieves rigidity of muscle , activates blood circulation and the channels. | n/a |
| Ban-Xia-Xie-Xin-Tang  半夏瀉心湯  Hangeshashinto  Banha-sasim-tang | *Pinellia ternata, Panax ginseng,*  *Scutellaria baicalensis,*  *Zingiber officinale(dried), Glycyrrhiza uralensis,*  *Ziziphus jujube,and*  *Coptis chinensis* | 1.Harmonizes the stomach and descends rebellious qi  2.Disperses accumulation, masses, and clumping.  3.Relieves fullness and focal distention. | n/a |
| Shao-Yao-Gan-Cao-Tang  芍藥甘草湯 | *Paeonia lactiflora and*  *Glycyrrhiza uralensis* | 1. Harmonizes qi and blood.  2.Releases spasm and relieving pain. | n/a |
| Long-Dan-Xie-Gan-Tang  龍膽瀉肝湯 | *Gentiana scabra,*  *Scutellaria baicalensis,*  *Gardenia jasminoides,*  *Alisma plantago,*  *Plantago asiatica,*  *Akebia trifoliate,*  *Rhemannia glutinosa,*  *Angelica sinensis,*  *Bupleurum chinense and Glycyrrhiza uralensis* | 1.Drains excess heat/fire from the liver and gallbladder.  2.Drains damp and clears heat from the lower burner. | n/a |
| Suan-Zao-Ren-Tang  酸棗仁湯 | *Ziziphi Spinosae*  *Semen,Anemarrhena,*  *Ligusticum striatum,*  *Poria cocos,and*  *Glycyrrhiza uralensis* | 1.Nourishes liver and heart blood nourishes yin. 2.Clears deficient heat and calms the spirit. | n/a |
| Xue-Fu-Zhu-Yu-Tang  血府逐瘀湯 | *Angelica sinensis (Oliv.) Diels.,*  *Rehmannia glutinosa (Gaert.) ,*  *Prunus davidiana (Carr.)Franch.,*  *Carthamus tinctorius,*  *Citrus aurantium L.,*  *Paeonia veitchii,*  *Bupleurum chinense ,*  *Glycyrrhiza uralensis,*  *Platycodonis,*  *Radix,Ligusticum striatum,and Achyranthes bidentata* | 1.Invigorates the blood and dispels blood stagnation.  2.Spreads liver qi, unblocks the channels, and relieves pain.. | Xue-Fu-Zhu-Yu-Tang  attenuates liver fibrosis via inhibiting angiogenesis.[29] |
| Xiang-Sha-Liu-Jun-Zi-Tang  香砂六君子湯 | *Rosa banksiae,*  *Amomum villosum,*  *Citrus reticulate Blanco,*  *Pinellia ternata,*  *Codonopsis pilosula, Atractylodes macrocephala,*  *Poria cocos,*  *Glycyrrhiza uralensis,*  *Zingiber officinale, and*  *Ziziphus jujube* | 1. Benefits stomach, invigorates spleen, and replenishes qi.  2. Regulates stomach qi-flowing. | n/a |
| Ping-Wei-San  平胃散 | *Atractylodes lancea,*  *Magnolia officinalis,*  *Citrus reticulata Blanco, Glycyrrhiza uralensis,*  *Zingiber officinale,and*  *Ziziphus jujube* | 1.Dries dampness and strengthens the spleen.  2.Activates and regulates the spleen and stomach qi.  3.Harmonizes the middle jiao. | n/a |

n/a: not available when searching with the formula pin-yin name or kampo name.

**References**

1. Z. Zhou, Y. Zhang, X.R. Ding, et al. "Protocatechuic aldehyde inhibits hepatitis B virus replication both in vitro and in vivo," *Antiviral Research, vol.* 74, no. 1, pp. 59-64.

2. Y. Xu, D. Feng, Y. Wang, S. Lin, and L. Xu. "Sodium tanshinone IIA sulfonate protects mice from ConA-induced hepatitis via inhibiting NF-kappaB and IFN-gamma/STAT1 pathways," *Journal of Clinical Immunology, vol.* 28, no. 5, pp. 512-519.

3. C. Jiang, W. Zhu, X. Yan, et al. "Rescue therapy with Tanshinone IIA hinders transition of acute kidney injury to chronic kidney disease via targeting GSK3β," *Scientific Reports, vol.* 6, p. 36698.

4. X. Xu, Y. Hu, X. Zhai, et al. "Salvianolic acid A preconditioning confers protection against concanavalin A-induced liver injury through SIRT1-mediated repression of p66shc in mice," *Toxicology and Applied Pharmacology, vol.* 273, no. 1, pp. 68-76.

5. H. Cai, S. Su, Y. Li, et al. "Protective effects of Salvia miltiorrhiza on adenine-induced chronic renal failure by regulating the metabolic profiling and modulating the NADPH oxidase/ROS/ERK and TGF-beta/Smad signaling pathways," *Journal of Ethnopharmacology, vol.* 212, pp. 153-165.

6. Y. Peng, T. Yang, K. Huang, L. Shen, Y. Tao, and C. Liu. "Salvia Miltiorrhiza Ameliorates Liver Fibrosis by Activating Hepatic Natural Killer Cells in Vivo and in Vitro," *Frontiers in Pharmacology, vol.* 9, p. 762.

7. H. Cai, S. Su, Y. Li, et al. "Danshen can interact with intestinal bacteria from normal and chronic renal failure rats," *Biomedicine and Pharmacotherapy, vol.* 109, pp. 1758-1771.

8. F. Yang, Y. Xu, A. Xiong, et al. "Evaluation of the protective effect of Rhei Radix et Rhizoma against alpha-naphthylisothiocyanate induced liver injury based on metabolic profile of bile acids," *Journal of Ethnopharmacology, vol.* 144, no. 3, pp. 599-604.

9. Z.H. He, R. Zhou, M.F. He, et al. "Anti-angiogenic effect and mechanism of rhein from Rhizoma Rhei," *Phytomedicine, vol.* 18, no. 6, pp. 470-478.

10. Y. Chen and J. Zhu. "Anti-HBV effect of individual traditional Chinese herbal medicine in vitro and in vivo: an analytic review," *Journal of Viral Hepatitis, vol.* 20, no. 7, pp. 445-452.

11. B. Dinda, S. Dinda, S. DasSharma, R. Banik, A. Chakraborty, and M. Dinda. "Therapeutic potentials of baicalin and its aglycone, baicalein against inflammatory disorders," *European Journal of Medicinal Chemistry, vol.* 131, pp. 68-80.

12. Y. Chen, Y. Zheng, Z. Zhou, and J. Wang. "Baicalein alleviates tubular-interstitial nephritis in vivo and in vitro by down-regulating NF-kappaB and MAPK pathways," *Brazilian Journal of Medical and Biological Research, vol.* 51, no. 10, p. e7476.

13. M. Lin, L. Li, L. Li, et al. "The protective effect of baicalin against renal ischemia-reperfusion injury through inhibition of inflammation and apoptosis," *BMC Complementary and Alternative Medicine, vol.* 14, no. 1, p. 19.

14. X.M. Meng, G.L. Ren, L. Gao, et al. "Anti-fibrotic effect of wogonin in renal tubular epithelial cells via Smad3-dependent mechanisms," *European Journal of Pharmacology, vol.* 789, pp. 134-143.

15. C.H. Park, M.R. Shin, B.K. An, et al. "Heat-Processed Scutellariae Radix Protects Hepatic Inflammation through the Amelioration of Oxidative Stress in Lipopolysaccharide-Induced Mice," *American Journal of Chinese Medicine, vol.* 45, no. 6, pp. 1233-1252.

16. H. Sun, Q.M. Che, X. Zhao, and X.P. Pu. "Antifibrotic effects of chronic baicalein administration in a CCl4 liver fibrosis model in rats," *European Journal of Pharmacology, vol.* 631, no. 1-3, pp. 53-60.

17. X. Li, X. Wang, C. Han, et al. "Astragaloside IV suppresses collagen production of activated hepatic stellate cells via oxidative stress-mediated p38 MAPK pathway," *Free Radical Biology and Medicine, vol.* 60, pp. 168-176.

18. L. Wang, Y.F. Chi, Z.T. Yuan, et al. "Astragaloside IV inhibits renal tubulointerstitial fibrosis by blocking TGF-beta/Smad signaling pathway in vivo and in vitro," *Experimental Biology and Medicine (Maywood, NJ), vol.* 239, no. 10, pp. 1310-1324.

19. T. Li, X.-h. Xu, Z.-h. Tang, et al. "Platycodin D induces apoptosis and triggers ERK- and JNK-mediated autophagy in human hepatocellular carcinoma BEL-7402 cells," *Acta Pharmacologica Sinica, vol.* 36, no. 12, pp. 1503-1513.

20. T. Li, Z.H. Tang, W.S. Xu, et al. "Platycodin D triggers autophagy through activation of extracellular signal-regulated kinase in hepatocellular carcinoma HepG2 cells," *European Journal of Pharmacology, vol.* 749, pp. 81-88.

21. S.E. Jin, Y.K. Son, B.S. Min, H.A. Jung, and J.S. Choi. "Anti-inflammatory and antioxidant activities of constituents isolated from Pueraria lobata roots," *Archives of Pharmacal Research, vol.* 35, no. 5, pp. 823-837.

22. R.W. Li, G. David Lin, S.P. Myers, and D.N. Leach. "Anti-inflammatory activity of Chinese medicinal vine plants," *Journal of Ethnopharmacology, vol.* 85, no. 1, pp. 61-67.

23. J. Xie, L. Guo, G. Pang, X. Wu, and M. Zhang. "Modulation effect of Semen Ziziphi Spinosae extracts on IL-1beta, IL-4, IL-6, IL-10, TNF-alpha and IFN-gamma in mouse serum," *Nat Prod Res, vol.* 25, no. 4, pp. 464-467.

24. S.C. Chien, W.C. Chang, P.H. Lin, et al. "A Chinese herbal medicine, jia-wei-xiao-yao-san, prevents dimethylnitrosamine-induced hepatic fibrosis in rats," *ScientificWorldJournal, vol.* 2014, p. 217525.

25. I. Shimizu, Y.R. Ma, Y. Mizobuchi, et al. "Effects of Sho-saiko-to, a Japanese herbal medicine, on hepatic fibrosis in rats," *Hepatology, vol.* 29, no. 1, pp. 149-160.

26. J. Li, R. Hu, S. Xu, et al. "Xiaochaihutang attenuates liver fibrosis by activation of Nrf2 pathway in rats," *Biomedicine and Pharmacotherapy, vol.* 96, pp. 847-853.

27. J.S. Chang, K.C. Wang, H.W. Liu, M.C. Chen, L.C. Chiang, and C.C. Lin. "Sho-saiko-to (Xiao-Chai-Hu-Tang) and crude saikosaponins inhibit hepatitis B virus in a stable HBV-producing cell line," *American Journal of Chinese Medicine, vol.* 35, no. 2, pp. 341-351.

28. M. Yamashiki, A. Nishimura, H. Suzuki, S. Sakaguchi, and Y. Kosaka. "Effects of the Japanese herbal medicine "Sho-saiko-to" (TJ-9) on in vitro interleukin-10 production by peripheral blood mononuclear cells of patients with chronic hepatitis C," *Hepatology, vol.* 25, no. 6, pp. 1390-1397.

29. Y.N. Zhou, M.Y. Sun, Y.P. Mu, et al. "Xuefuzhuyu decoction inhibition of angiogenesis attenuates liver fibrosis induced by CCl(4) in mice," *Journal of Ethnopharmacology, vol.* 153, no. 3, pp. 659-666.
